# Discovering Implicit Entity Relation with the Gene-Citation-Gene Network

**DOI:** 10.1371/journal.pone.0084639

**Published:** 2013-12-17

**Authors:** Min Song, Nam-Gi Han, Yong-Hwan Kim, Ying Ding, Tamy Chambers

**Affiliations:** 1 Department of Library and Information Science, Yonsei University, Seoul, Republic of Korea; 2 Department of Information and Library Science, Indiana University, Bloomington, Indiana, United States of America; Wake Forest School of Medicine, United States of America

## Abstract

In this paper, we apply the entitymetrics model to our constructed Gene-Citation-Gene (GCG) network. Based on the premise there is a hidden, but plausible, relationship between an entity in one article and an entity in its citing article, we constructed a GCG network of gene pairs implicitly connected through citation. We compare the performance of this GCG network to a gene-gene (GG) network constructed over the same corpus but which uses gene pairs explicitly connected through traditional co-occurrence. Using 331,411 MEDLINE abstracts collected from 18,323 seed articles and their references, we identify 25 gene pairs. A comparison of these pairs with interactions found in BioGRID reveal that 96% of the gene pairs in the GCG network have known interactions. We measure network performance using degree, weighted degree, closeness, betweenness centrality and PageRank. Combining all measures, we find the GCG network has more gene pairs, but a lower matching rate than the GG network. However, combining top ranked genes in both networks produces a matching rate of 35.53%. By visualizing both the GG and GCG networks, we find that cancer is the most dominant disease associated with the genes in both networks. Overall, the study indicates that the GCG network can be useful for detecting gene interaction in an implicit manner.

## Introduction

The proliferation of digitized biomedical literature and other resources has opened new avenues for both researchers and practitioners; yet, the effective use of these multi-heterogeneous resources remains a fundamental issue [1]. Scientific articles contain various entities, including author, journal, institute, country, topic, keyword, method, domain, etc., which Ding et al. [2] divided into two types: evaluative and knowledge. Evaluative entities are those traditionally used to measure scholarly impact [3], such as paper, author, journal, institution, and country. Knowledge entities are those individual bits of knowledge extracted from the scientific text, such as keyword, dataset, key method, key theory, gene, drug, or disease; the extraction and the subsequent analysis of which has the potential to lead to new hypothesis and knowledge [2]. However, in an era of big biomedical data, discovery of these hidden relationships between biomedical knowledge entities and the resulting generation of new hypotheses is both a goal and a challenge. 

Many studies have sought to detect these hidden relationships believed buried in large unstructured biomedical text collections [4] using an entity co-occurrence approach [5–15], which assumes there exists a relationship between two entities if both appear within the same document. In fact, analysis of knowledge entities using co-occurrence, has been successfully used in the biomedical field for more than a decade when Strapley et al. [5] first constructed and analyzed a gene-gene network based on gene pairs extracted from Medline records indexed with the mesh term ‘Saccharomyces cerevisiae’. That 2000 study concluded, while gene clusters rad50, MRE11, and xrs2 belong to DNA double-stranded break repair and gene clusters RAD27, DHS1, and DIN7 belong to DNA mismatch repair, they none-the-less have a co-occurrence relationship.

Subsequent studies have used similar co-occurrence relationships to build entity networks to examine different accuracy comparisons and measures [6–11] or different text mining algorithms and techniques [12–15]. However, the recently proposed entitymetrics approach [2], based on the assumption there exists a topical relationship between two articles when one cites the other, uses an entity network to discover new knowledge. The entitymetrics model contends there is a hidden, but plausible, relationship between an entity in one article and an entity in its citing article, and that analysis of the relationship between those entities can leads to knowledge discovery and a better understanding of knowledge acquisition.

In this study, we apply the entitymetrics model to a Gene-Citation-Gene (GCG) network constructed with gene pairs implicitly connected through citation. The advantage of the GCG network is its ability to detect implicit relations between entities, otherwise excluded, but which, none-the-less have an important interactive relationship. To evaluate this advantage, we compared the performance of our GCG network to a traditional Gene-Gene (GG) network using co-occurring entities from the same paper and covering the same corpus. Based on previous studies [2,11], our evaluation included the following network measures: degree, weighted degree, closeness, betweenness centrality, and PageRank. We calculated the top 25 gene pairs for each measure and compared each against known gene interactions identified in BioGRID. For gene pairs not found, we conducted a literature review to identify novel gene pairs not previously reported in the literature. Additionally, because of computational complexity, measures for ranking co-occurrences often use window size, shape, and distance metrics[16] based on a small (about 100) subset of words, however in this study we have used all words from the data collection to ensure dimension selection is based on the data and not on human judgment or simple word frequencies. 

We have organized the rest of this paper as follows: The Methodology section describes our data collection, gene entity identification, gene-gene pair and gene-citation-gene pair network construction, and an analysis of both networks. The Results and Discussion section describes results for the top 25 gene pairs based on co-occurrence frequency for each network, the results for the top 25 gene pairs based on network analysis measures for each network, the results for the top 25 gene pairs based on all measures, and results of a visualization analysis of each network. The Conclusion section summarizes our results, offers conclusions of based on the current work, and proposes future work. 

## Methodology

### Data Collection

To provide broader coverage than previous studies, we chose to build a customized citation database using a set of bioinformatics seed articles and their reference lists instead of mining based on keywords or over a specific time-period, which would have confined the results to select journals or subject fields. We identified seed articles from bioinformatics related journals, using selection criteria based on a study by Huang et al. [17]. Although we used most of the journals identified in their research, we also added journals identified from the International Society of Computational Biology publications list (http://www.iscb.org/iscb-publications-journals), Wikipedia’s bioinformatics journal list (http://en.wikipedia.org/wiki/List_of_bioinformatics_journals), and the Web of Science’s Science Journal Citation Reports (SJCR). After excluding journals with less than 200 citations, our dataset included 18,323 seed articles from 48 journals.

**Figure 1:**
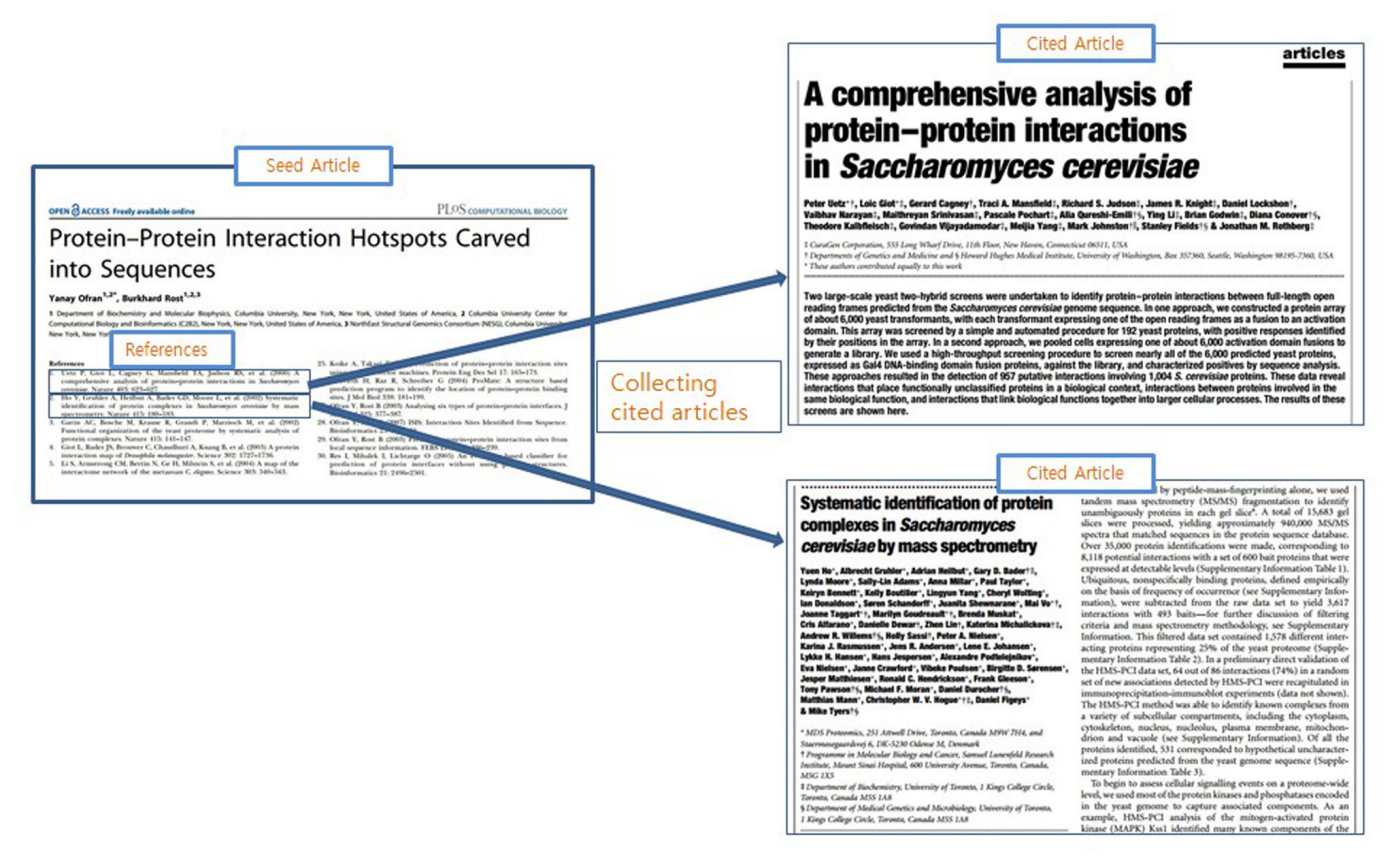
Overall procedure for creating the Gene-Citation-Gene Network.

**Figure 2:**
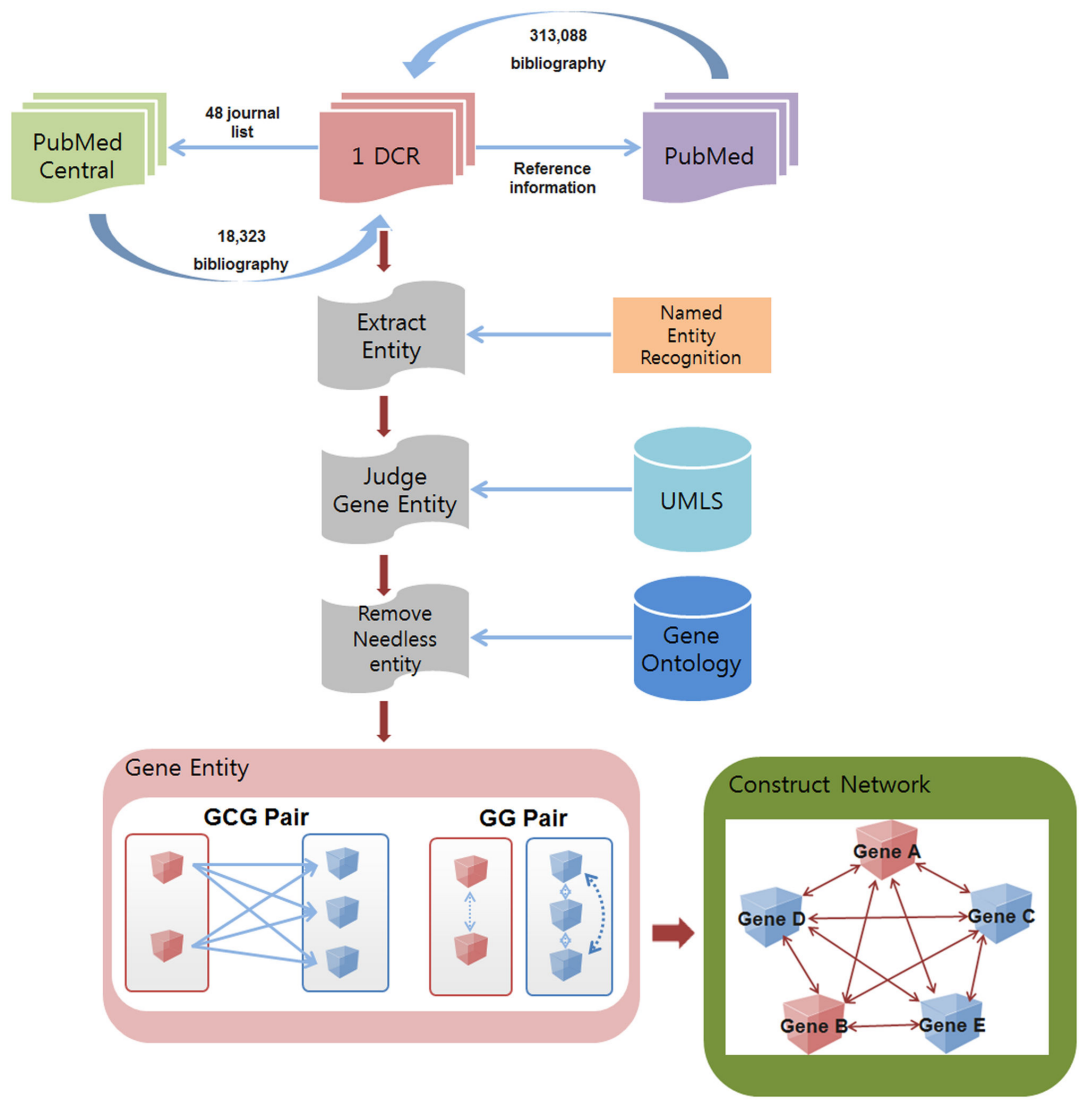
Example of title and reference list used in creation of 1DCR database.

After which, we parsed the full-text of each article and their reference lists stored in XML format using an automatic procedure written in JAVA. Using the reference lists of each article, we then queried PubMed to collect abstract information for each reference based on title and stored this data in a MySQL database. Figure 1 shows an example title and the references used in our dataset. This procedure (see Figure 2) resulted in the collection of 313,088 additional abstracts. Combined with the original 18,323 seed articles, our 1DCR (1-Depth Citation Relationship) DB contained bibliographic information for 331,411 abstracts based on 1-depth citation data between an article and its reference list. 

### Gene Entity Identification

We extracted gene pairs from the 331,411 abstracts using the Conditional Random Field (CRF)-based Named Entity Recognition (NER) technique developed by McCallum and Wei [18] and filtered the extracted entities based on comparison with Unified Medical Language System (UMLS). As UMLS classifies entities by semantic type, our study extracted entities only if the semantic type was gene or genome. We used the UMLS Concept Unique Identifiers (CUI), to identify a preferred term for each extracted entity, which allowed for the merger of similar terms and synonyms. Using GO (gene ontology) we further filtered terms pertinent to genes such as *genes*, *genomes*, or *alleles* but not gene itself. Out of 331,411 articles, 118,151 had matching GO terms resulting in 9,940 uniquely identified genes (Table 1). 

**Table 1:** Article and Entity Statistics.

Articles	Articles that contain *Gene* or *Genome* Entity	*Gene* or *Genome* Entities	*Gene* or *Genome* Entities Filtered by CUI and GO
331,411	118,151	558,705	9,940

### Gene-Gene Pair and Gene-Citation-Gene Pair

**Figure 3:**
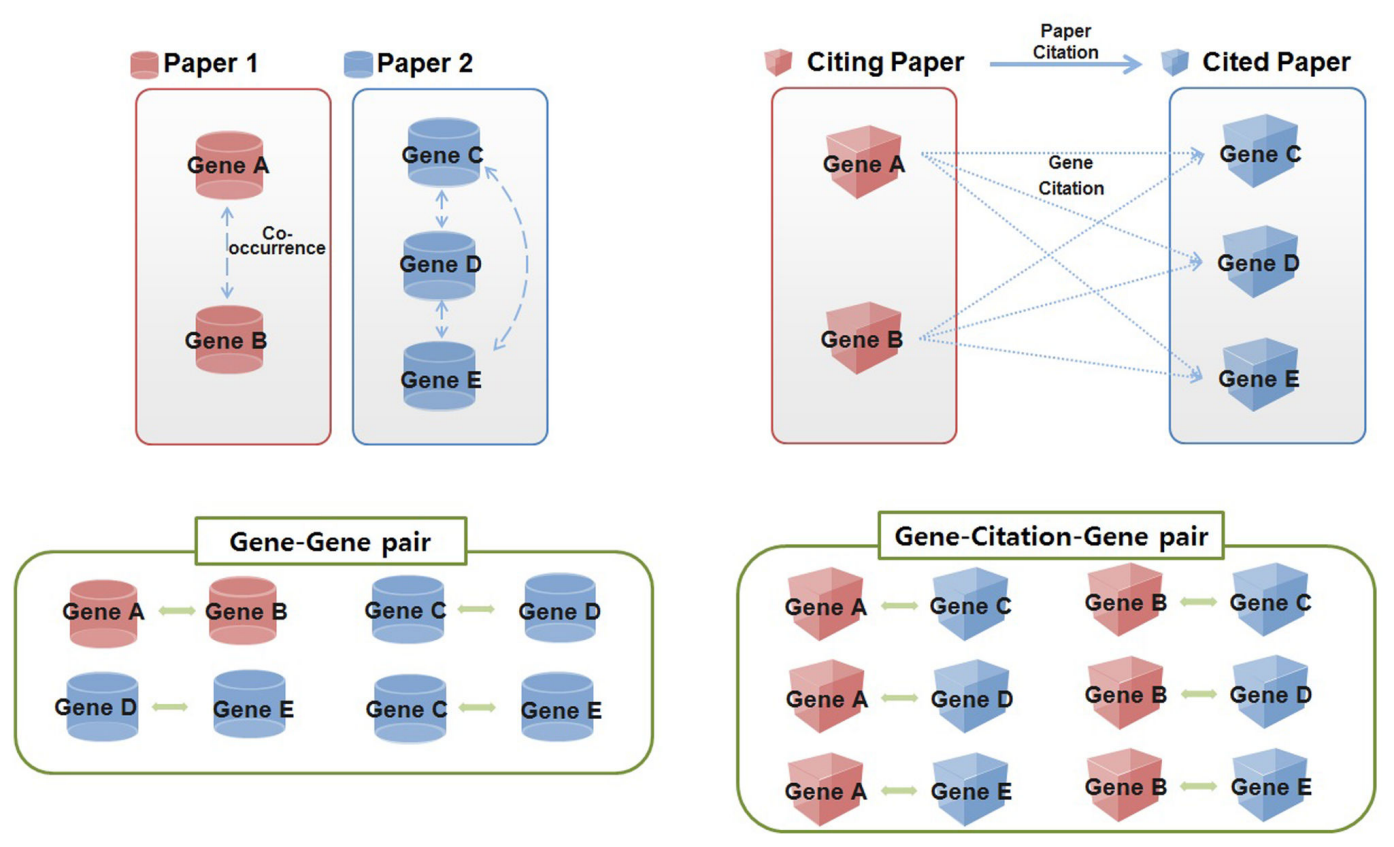
Two types of pairs: Gene-Gene pair and Gene-Citation-Gene pair.

Based on the identified genes, we constructed two types of gene pairs: gene-gene pairs and gene-citation-gene pairs. We constructed gene-gene pairs based on co-occurrence within the same article and calculated a co-occurrence frequency for each. We constructed the gene-citation-gene pairs based on the implicit linkage between genes in one article and genes in a cited article and calculated a co-occurrence frequency for each pair. Despite the directional nature of a citation, we did not consider directionality when identifying the gene-citation-gene pairs for this study. Given our focus on identifying a relationship between genes, the directionality of citation provided no additional understanding as it might not reflect gene-to-gene directionality. By repeating this pairing procedure (Figure 3), we built both a gene-gene network and a gene-citation-gene network.

### Network Analysis

We used Gephi [19], an open source social network analysis tool, to analyze and visualize the created networks. To understand the core relationships in each network, we selected gene pairs using co-occurrence frequency and identified core nodes using centrality, similar to previous studies [4,11,20,21]. In those studies, Estrada [20] identified factors influencing node centrality, such as, node degree of directly interacting nodes (genes), node closeness, and the quantity of node pairs requiring a specific intermediary node for communications, Goh et al. [4] and Hahn and Kern [21] both identified an association between betweenness centrality and the essentiality of a gene, and Ozgur et al. [11] found that genes centrally located in the disease-specific network were related to the disease. 

To understand specifically which genes play a major role in the field of bioinformatics, we identified the important nodes (top-ranked genes) in both the GCG and GG network using the network measures degree centrality, weighted degree centrality, closeness centrality, betweenness centrality, and PageRank. After which, we confirmed that each top-ranked gene pair existed in the Biological General Repository for Interaction Datasets (BioGRID) database [22] and then queried BioGRID to identify a gene interaction list of gene for comparison. 

We identified the characteristics of individual genes using the NCBI Gene DB (http://www.ncbi.nlm.nih.gov/gene/) and GeneCards (http://www.genecards.org/). The NCBI Gene DB includes primarily genomes related data, such as gene products and their attributes (e.g., protein interactions), associated markers, phenotypes, interactions, and links to citations. GeneCards focuses on human genes data such as, gene related transcriptomic, genetic, proteomic, functional, and disease information.

## Results and Discussion

### Top 25 Gene Pairs Based on Co-Occurrence Frequency

Using co-occurrence frequency, we can identify frequently co-occurring genes in bioinformatics. Assuming that frequently occurring genes represent the core genes in bioinformatics, we can then use network analysis to gain insight into how these genes interact with each other. 

Table 2 shows the top 25 gene pairs by co-occurrence frequency for both the GG network and the GCG network. There is a clear difference in gene rank order between two networks. Of the 38 gene pairs identified across the two networks, only 12 appear in both networks. We examined the correlation between top 25 pairs from the GG network and the GCG network by spearman rho and did not find a significant correlation (p value = 0.402. p < 0.01). In general, gene pairs tend to have higher co-occurrence frequency in the GCG network; of the 12 overlapping gene pairs, seven have a higher frequency in the GCG network. 

**Table 2:** Top 25 gene pairs by frequency.

**Gene-Gene Network**			**Gene-Citation-Gene Network**		
*Gene*	*Freq*	*Remarks*	*Gene*	*Freq*	*Remarks*
MDM2-TP53*	6684	Interaction	MDM2-TP53*	7264	Interaction
POU5F1-SOX2*	3116	Interaction	TP53-TP63*	2799	Interaction
NANOG-POU5F1*	2805	Interaction	TP53-TP73*	2686	Interaction
NANOG-SOX2	1912	Interaction	PARK2-PINK1*	2521	Interaction
PARK2-PINK1*	1726	Interaction	CDC20-MXI1*	2507	Interaction
DMC1-RAD51*	1671	Interaction	CDKN2A-TP53*	1963	Interaction
HRAS-TP53*	1649	Interaction	MDM4-TP53	1845	Interaction
TP53-TP73*	1604	Interaction	DISC1-NDEL1	1778	Interaction
TP53-TP63*	1555	Interaction	MDM2-MDM4*	1553	Interaction
DNMT3A-DNMT3B	1471	Interaction	NEDD4-NEDD4L	1461	Same gene family
CDC20-MXI1*	1392	Interaction	CTCF-CTCFL	1389	Same gene family
RAD51-RAD52	1301	Interaction	POU5F1-SOX2*	1320	Interaction
CDKN2A-TP53*	1225	Interaction	TP53-USP7	1283	Interaction
DNMT3A-DNMT3L	1139	Interaction	BCL2-BCL2L1	1199	Interaction
ARNTL-CLOCK*	1069	Interaction	BCL2-SOD1	1112	Interaction
SMN1-SMN2	868	Interaction	DMC1-RAD51*	1074	Interaction
MDM2-MDM4*	840	Interaction	TP63-TP73	907	Interaction
RAG1-RAG2	816	Interaction	HRAS-TP53*	904	Interaction
MRE11A-RAD50	814	Interaction	ECT2-PLK1	884	Confirm interaction with literature review
MXD1-MXI1	763	Same gene family	DISC1-NDE1	858	Confirm interaction with literature review
ATR-CHEK2	751	Interaction	NANOG-POU5F1*	851	Interaction
BCL2-TP53	727	Interaction	ARNTL-CLOCK*	850	Interaction
BUB1-BUB1B	702	Interaction	MDM2-USP7	822	Interaction
CTCF-H19	676	Confirm interaction with literature review	FXR1-FXR2	815	Interaction
DIO_2_-LMOD1	669	Same gene family	SOX9-WNT4	810	No interaction

Gene pairs in bold print appear in both networks.

When we compared our results against known interactions from BioGRID, we identified 22 of 25 gene pairs from the GG network were reported and 20 of 25 gene pairs from the GCG network were reported. Of the eight gene pairs not reported in BioGRID, four (CTCF-CTCFL, DIO_2_-LMOD1, MXD1-MXI1, NEDD4-NEDD4L) were not found because they belonged to the same gene family and therefore tend to co-occur frequently and three (CTCF-H19, ECT2-PLK1DISC1-NDE1) were confirmed to have interactions based on literature review using PubMed. In the literature review we identified papers, which reported an interaction between the two genes, though explicit co-occurrence in the title or the abstract. Table 3 lists articles reporting interactions for the three gene pairs.

**Table 3:** Articles reporting findings for gene pairs confirmed through literature review.

***CTCF-H19^1^***	***DISC1-NDE1^2^***	***ECT2-PLK1^2^***
Grbesa *et al*.[34].	Bradshaw *et al*.[37].	Li *et al.*[40]
Tost *et al*.[35].	Moens *et al*.[38].	Wolfe *et al*.[41].
*De Castro Valente* *Esteves et al* [36].	Burdick *et al*.[39].	*Niiya et al* [42].

^1^ CTCF-H19 was identified only by the gene-gene network.

^2^ ECT2-PLK1 and DISC1-NDE1 were identified only by the gene-citation-gene network.

Excluding the same family gene pairs, we confirmed that 88% of the gene pairs identified in the GG network have a known interaction and that 80% of pairs identified in the GCG network have a known interaction. Including, the same family genes pairs (which are in fact interaction pairs), we confirmed that all top 25 gene pairs identified in the GG network have a known interaction whereas 96% of the top 25 gene pairs identified in the GCG network have a known interaction. In the GG network, top 1.19% of pairs co-occurring 100 times or more (663 pairs) represent 30% of the total number of pair frequencies. In the GCG network, top 0.81% of pairs co-occurring 100 times or more (632 pairs) represent 23% of the total number of pair frequencies. Analyzing all pairs of both networks reveal 13,749 pairs common to both networks, which represents about 11.5% of all pairs. In terms of frequency, pairs commonly appearing in both networks represent 55% of the GG network and 49% of the GCG network. The fact that top gene pairs by frequency represent a high proportion in all gene pairs indicates that the highly ranked gene pairs by frequency in both networks are those gene pairs commonly appearing in both networks. This observation is confirmed using the Spearman rho test which identifies a significant correlation (correlation coefficient = 0.364, p< 0.01) between the rank of pairs commonly appearing in both networks. This implies that top gene pairs commonly appearing in both networks are significant in bioinformatics. 

### Top 25 Genes Based on Network Analysis Measures

We calculated the top 25 nodes using each of the following measures: degree centrality, weighted degree centrality, closeness centrality, betweenness centrality, and PageRank. Except for closeness centrality and PageRank, we included only top 25 nodes with a weight of 10 or higher because of the high number of nodes with tie weights. 

The degree centrality of a node denotes the number of links that node has with other nodes. Betweenness centrality is the number of shortest paths passing through a node. Nodes with a high betweenness centrality serve as bridges connecting different sub-groups. PageRank measures the importance of a node based on the sum of the rank of its backlinks (the number of nodes that link to that particular node). Since these three network measures resulted in the identification of similar nodes, we focus on weighted degree for analysis and closeness to show unique results. Tables A-C in the Appendix S1 show results for degree centrality, betweenness centrality and PageRank.. 

We display all results with the associated disease categories based on the Genetic Association Database (GAD). In GAD there may be several diseases associated with a given gene [23], however, for this study, we used only the most dominant disease and used the GAD taxonomy to simplify identification to a specific disease category. The disease categories provided by GAD include AGING, CANCER, CARDIOVASCULAR, CHEMDEPENDENCY, DEVELOPMENTAL, HEMATOLOGICAL, IMMUNE, INFECTION, METABOLIC, MITOCHONDRIAL, NEUROLOGICAL, NORMAL VARIATION, OTHER, PHARMACOGENOMIC, PSYCHIATRIC, RENAL, REPRODUCTION, UNKNOWN, and VISION.

### Weighted Degree Centrality

Weighted degree centrality is a variation of degree centrality calculated by summing the frequency of every node pair for a given node. Table 4 shows the top 25 gene pairs by weighted degree centrality and GAD disease category. We note that 16 of the top 25 genes (64%) in the GG network and 14 (56%) in the GCG network are related to cancer. Of the top 25 genes, 64% occur in both networks.

**Table 4:** Top 25 genes by weighted degree centrality and associated GAD disease category.

Gene-Gene Network			Gene-Citation-Gene Network		
*Gene*	*Degree*	*Disease Category*	*Gene*	*Degree*	*Disease Category*
TP53	23090	CANCER ( 360 )	TP53	29859	CANCER ( 360 )
MDM2	8451	CANCER ( 126 )	MDM2	11413	CANCER ( 126 )
POU5F1	7187	IMMUNE ( 10 )	RAD51	4991	CANCER ( 68 )
SOX2	6278	VISION ( 5 )	PINK1	4974	NEUROLOGICAL ( 39 )
CLOCK	5145	PSYCH ( 16 )	TP63	4717	CANCER ( 11 )
NANOG	4808	CARDIOVASCULAR ( 1 )	CLOCK	4536	PSYCH ( 16 )
RAD51	4584	CANCER ( 68 )	SOX2	4244	VISION ( 5 )
MXI1	3397	IMMUNE ( 2 )	MXI1	4099	IMMUNE ( 2 )
MYC	3275	CANCER ( 27 )	CDC20	3882	CANCER ( 2 )
DNMT3A	3063	CANCER ( 7 )	TP73	3786	CANCER ( 28 )
CDC20	2810	CANCER ( 68 )	PARK2	3735	NEUROLOGICAL ( 82 )
PINK1	2569	NEUROLOGICAL ( 39 )	MDM4	3630	CANCER ( 6 )
DMC1	2476	REPRODUCTION ( 2 )	CTCF	3475	METABOLIC ( 3 )
HRAS	2420	CANCER ( 25 )	MYC	3466	CANCER ( 27 )
CDKN2A	2393	CANCER ( 131 )	BCL2	3203	CANCER ( 33 )
DNMT3B	2357	CANCER ( 27 )	DISC1	3133	PSYCH ( 38 )
E2F1	2340	CANCER ( 2 )	NDEL1	3121	PSYCH ( 3 )
TP73	2323	CANCER ( 28 )	CDKN2A	3048	CANCER ( 131 )
TP63	2252	CANCER ( 11 )	POU5F1	2998	IMMUNE ( 10 )
BCL2	2197	CANCER ( 33 )	DMC1	2549	REPRODUCTION ( 2 )
BUB1B	2045	CANCER ( 3 )	PLK1	2505	CANCER ( 6 )
PARK2	2041	NEUROLOGICAL ( 82 )	CTCFL	2463	CANCER ( 1 )
ATR	2021	CARDIOVASCULAR ( 2 )	BCL2L1	2373	CANCER ( 7 )
CHEK2	1992	CANCER ( 105 )	USP7	2309	CARDIOVASCULAR ( 2 )
RAD52	1808	CANCER ( 19 )	RAD9A	2306	CANCER ( 1 )

Numbers displayed in parenthesis showing the number of papers that report association between the given gene and a disease.

Important genes, actively researched in the biomedical domain, identified in Table 3 includeTP53, MDM2, POU5F1, SOX2, CLOCK, RAD51, and PINK. Review of these genes using the NCBI Gene DB and GeneCards reveals that the TP53 gene encodes the tumor suppressor protein reported in cancer related papers and is in the same family as TP63, the MDM2 gene encodes proteins to promote tumor formation and is associated with cancer, the POU5F1 gene relates to embryonic stem cells, the SOX2 gene sustains stem cells associated with embryonic development and cell fate, the CLOCK gene plays a role in circadian rhythm and metabolism, RAD51 gene provides homologous recombination and repair of DNA, and the PINK1 gene relates to mitochondria. 

### Closeness Centrality

Closeness centrality, unlike degree centrality, focuses on the nodes extensibility of influence over the entire network. Table 5 shows the closeness value of nodes, calculated using the Brandes’ algorithm [24]. The top 25 genes, identified by closeness centrality, differ from those identified using the other measures. In the GG network, many of the identified genes relate to metabolic disease (32%), whereas in the GCG network most genes relate to a variety of diseases.

**Table 5:** Top 25 genes by closeness centrality and associated GAD disease category.

**Gene-Gene Network**			**Gene-Citation-Gene Network**		
*Gene*	*Closeness*	*Disease Category*	*Gene*	*Closeness*	*Disease Category*
MC4R	10.22680412	**METABOLIC ( 95 )**	SH2B3	9.095982143	**IMMUNE ( 18 )**
EIF4G1	10.1443299		OLIG2	9.095982143	**PSYCH ( 3 )**
TRAF3IP1	9.664948454		BLCAP	9.080357143	
MAP1A	9.664948454	**PSYCH ( 2 )**	BMP4	8.631696429	**CANCER ( 7 )**
PIF1	9.412371134		RPGRIP1L	8.595982143	**PSYCH ( 1 )**
FEN1	9.412371134	**CANCER ( 5 )**	RCC1	8.595982143	
CDKAL1	9.231958763	**IMMUNE ( 13 )**	RAB3IP	8.595982143	
SLC30A8	9.231958763	**METABOLIC ( 88 )**	RAB8A	8.59375	
IGF2BP2	9.231958763	**METABOLIC ( 78 )**	CEP290	8.59375	
FTO	9.229381443	**METABOLIC ( 197 )**	ALKBH1	8.495535714	
NPAT	9.146907216		KDM5A	8.495535714	
NDE1	8.677835052	**PSYCH ( 3 )**	KDM4A	8.495535714	**CARDIOVASCULAR ( 1 )**
NDEL1	8.670103093		KDM4C	8.495535714	
DISC1	8.667525773	**PSYCH ( 38 )**	JARID2	8.495535714	
CYBB	8.590206186		RPH3A	8.457589286	
OTX1	8.50257732		RPS6KB1	8.354910714	**CANCER ( 2 )**
CHL1	8.425257732	**HEMATOLOGICAL ( 2 )**	NPR1	8.354910714	**CARDIOVASCULAR ( 12 )**
		**PSYCH ( 3 )**			
DNA2	8.414948454	**CHEMDEPENDENCY ( 1 )**	RICTOR	8.354910714	**CANCER ( 1 )**
KCNE1	8.270618557	**CARDIOVASCULAR ( 37 )**	NPRL2	8.354910714	
HHEX	8.244845361	**METABOLIC ( 92 )**	NPR2	8.354910714	**CARDIOVASCULAR ( 2 )**
MSX2	8.18814433	**METABOLIC ( 5 )**	SPSB1	8.332589286	
NEUROG1	8.167525773	**PSYCH ( 4 )**	SPSB2	8.332589286	
MEF2C	8.164948454	**METABOLIC ( 7 )**	SPSB4	8.332589286	
MYOG	8.164948454	**METABOLIC ( 1 )**	KDM6A	8.207589286	**IMMUNE ( 1 )**
ELSPBP1	8.154639175		IRS2	8.178571429	**METABOLIC ( 29 )**

Important genes, actively researched in the biomedical domain, identified in Table 4 include MC4R, EIF4G1, TRAF3IP1, MAP1A, PIF1, SH2B3, OLIG2, BLCAP, BMP4, and RPGRIP1L. Review of these genes using the NCBI Gene DB and GeneCards reveals the MC4R gene produces membrane-bound receptors and relates to melanin cells, the EIF4G1 gene produces multi-subunit proteins, which constructs complex EIF4F related to mRNA activities, the TRAF3IP1 gene plays a secondary role in binding DNA and activates other genes, the MAP1A gene relates to neurogenesis, the PIF1 gene is a DNA helicase, the SH2B3 gene encodes proteins that play an important role in hematopoiesis, OLIG2 gene relates to oligodendroglial tumors of the brain, BLCAP gene encodes a tumor suppressor protein, the BMP4 gene encodes a bone morphogenetic protein, and the RPGRIP1L gene relates to genetic diseases. Defects in the RPGRIP1L gene causes Joubert syndrome type 7 (JBTS7) and Meckel syndrome type 5 (MKS5).

### Top 25 Genes Based on All Measures

**Figure 4:**
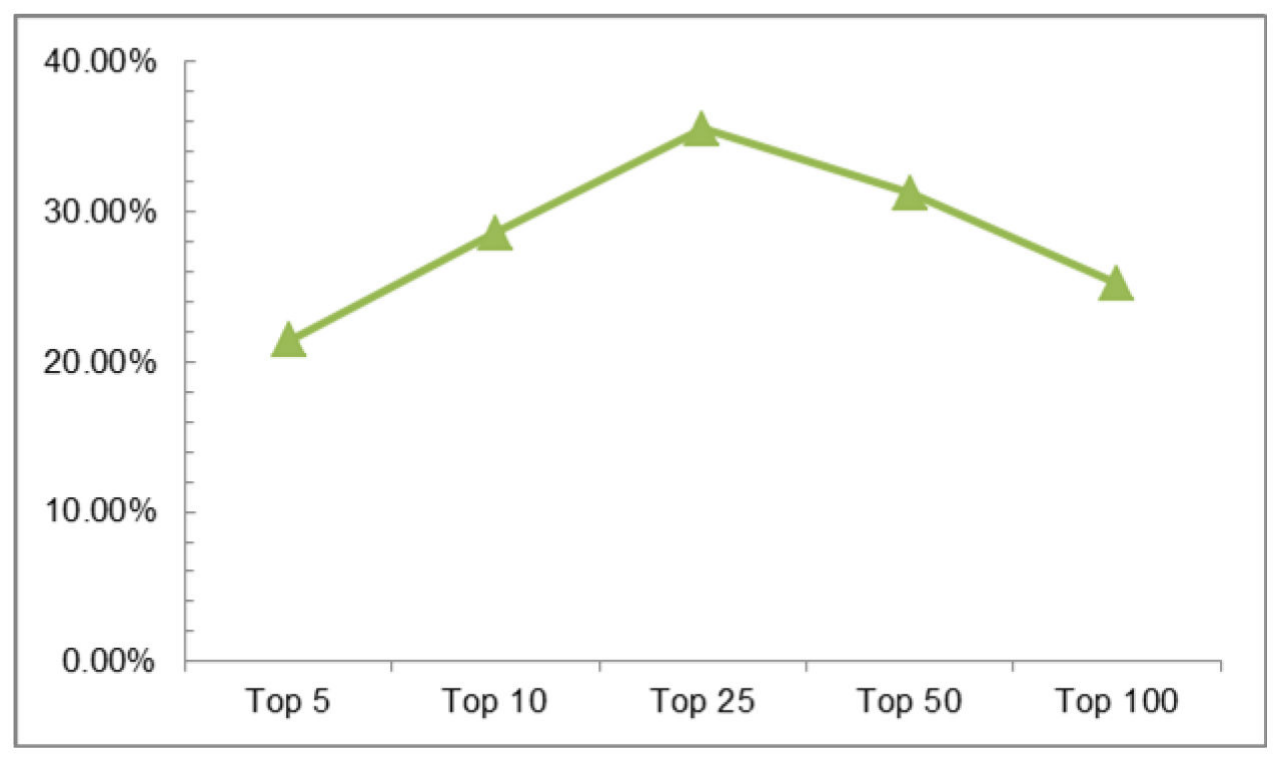
Matching rate with BioGRID according to top ranked node.

When we combined the top 25 ranked nodes by all measures for each network, we identified 239 gene pairs in the GG network and 303 gene pairs in the GCG network. Comparison of these networks against BioGRID revealed 67 pairs (28.03%) in the GG network and 55 pairs (18.15%) in the GCG network matched known interactions. The GCG network, while identifying more gene pairs, none-the-less had a lower matching rate than the GG network. To examine the accuracy of combined pairs of genes from both networks, we identified gene pairs appearing in both networks and compared them against BioGRID. Of the 76 gene pairs appearing in both networks, 27 matched with BioGRID (35.53%). Using the same process, we analyzed gene pairs appearing in both networks based on the top 5, 10, 50, and 100 ranked genes to reveal accuracy rates of 21.43%, 28.57%, 31.28%, 25.26% respectively (Figure 4).

### Visualization Analysis of Network

**Figure 5:**
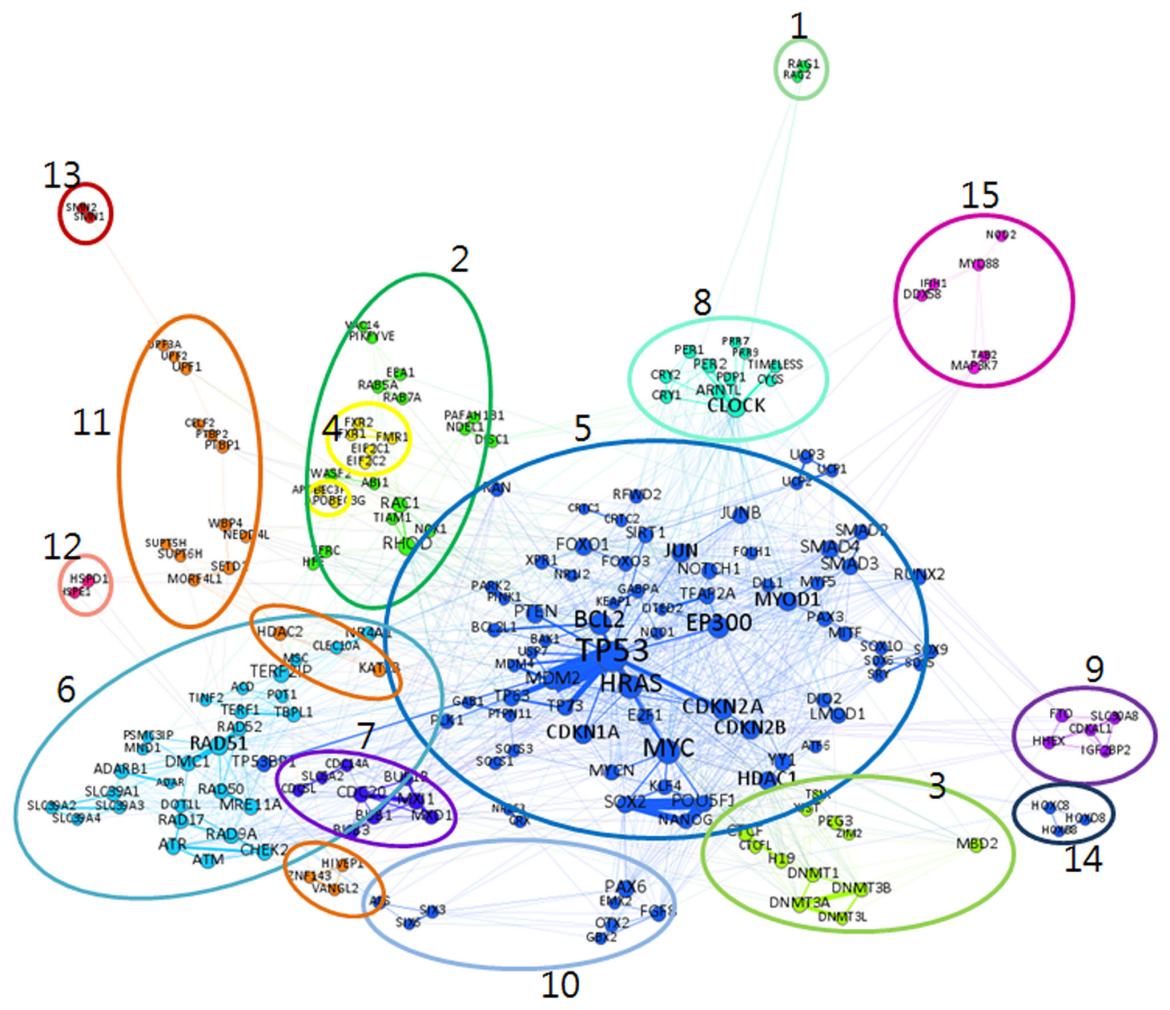
Visualization of Gene-Gene Network. Cluster 1: Immunologic deficiency and Lymphoma diseases; Cluster 2: Various diseases including tobacco use disorder, amyotrophic lateral sclerosis, and schizophrenia; Cluster 3: Breast and epithelial ovarian cancers; Cluster 4: Kidney, oral, and esophageal diseases; Cluster 5: Various diseases including breast and lung cancers and neoplasms; Cluster 6: Brain or nerve related disease including meningioma and cancer; Cluster 7: Cancer and neurological disease; Cluster 8: Prostate cancer and neurological diseases including depression, schizophrenia; Cluster 9: Calcinosis, HIV, obesity, and diabetes diseases; Cluster 10: Eye; Cluster 11: Various diseases including tobacco use disorder and schizophrenia; Cluster 12: Rheumatoid arthritis disease; Cluster 13: Muscular atrophy disease; Cluster 14: Clubfoot and bone mineral density diseases; Cluster 15: Autoimmune disease.

A visual analysis of the GG network (Figure 5) shows 15 clusters grouped by the modularity algorithm[25] . The modularity algorithm identifies groups of nodes in a network, which are more similar to each other than to other groups and optimizes the detection the community structure in networks. Each cluster identifies dominant diseases and representative genes belonging to that cluster. The Figure 5 caption identifies the disease associated with each cluster. In general, the GG network shows broadly spread genes associated first with cancer and then with neurology related diseases. Cluster 1, 9, and 13 are small clusters that have common diseases associated with most of representative genes. Cluster 10 has genes associated with eye related diseases, and cluster 6 has genes related to cancer. In the other clusters, there is no one single dominant disease but rather a mixture of various diseases.

**Figure 6:**
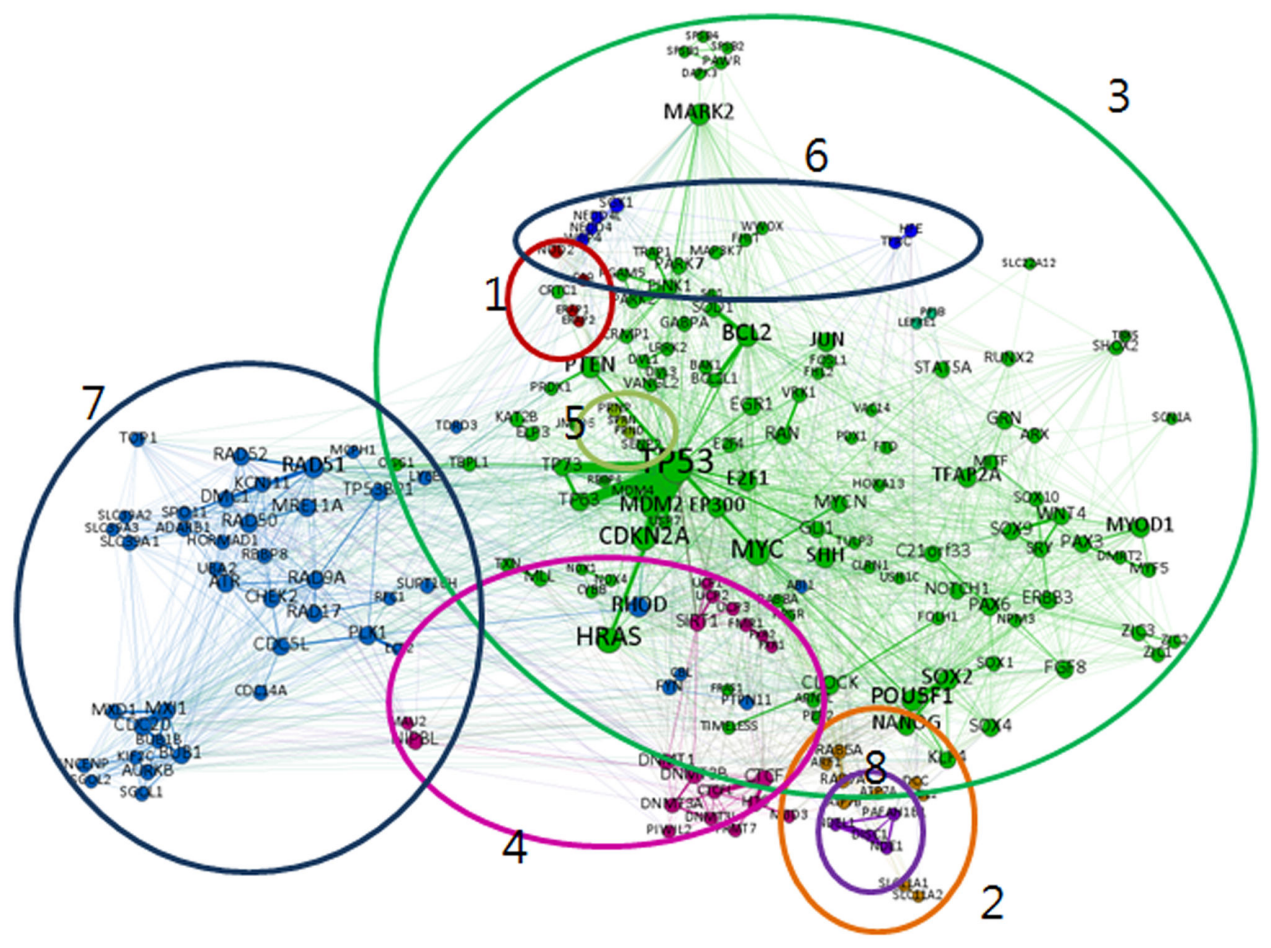
Visualization of Gene-Citation-Gene Network. Cluster 1: Pre-eclampsia and spondylitis diseases; Cluster 2: Various diseases including tuberculosis, abortion, and spontaneous; Cluster 3: Cancer and neoplasms diseases; Cluster 4: Obesity and various cancers; Cluster 5: Brain related diseases including Creutzfeld-Jakob and Alzheimer's disease; Cluster 6: Blood related diseases and heart failure; Cluster 7: Various cancers; Cluster 8: Schizophrenia.

A visualization of the GCG network (Figure 6) shows eight major clusters grouped by the modularity algorithm. The Figure 6 caption identifies the disease associated with each cluster. In the GCG network, there are fewer diseases commonly appearing in clusters than in the GG network. The small size clusters such as cluster 1, 5, 6, and 8, display a small number of common diseases. Frequently appearing diseases in these clusters are pre-eclampsia and spondylitis diseases (cluster 1), brain (cluster 5), blood related disease and heart failure (cluster 6), and schizophrenia (cluster 8). 

Table D and E in the Appendix S1 identifies the representative genes and diseases associated with each cluster in both the GG and GCG networks. Using GAD, we identified the appropriate disease associated with each cluster based on the majority of genes in each cluster. GAD collects, standardizes, and archives genetic associated data [23]. We selected salient diseases by the number of genes studied for a particular disease over the number of total genes in the cluster. For example, in cluster 1 in Table D, we chose Lymphoma (Non-Hodgkin) disease because there are two genes in cluster 1 and both genes mentioned the disease. Among the selected diseases, we label each cluster with the commonly mentioned diseases.

Using GAD, we can also confirm that there are articles studying the relationship between the genes in a specific cluster and a certain disease. In particular, we observed that cancer is a dominant disease that is associated with a wide range of genes in both the GG and GCG networks, 15 clusters in the GG and eight in the GCG network. 

Overall the GG network consists of various sized clusters, while the GCG network contains two major and six minor clusters. Each network displays diseases differently as well; in the GG network five clusters relate to cancer, whereas in the GCG network cancer related genes form two large clusters with other disease related genes appearing in small clusters. In other words, the GG network shows that a set of genes with similar properties tend to form a fragmented cluster, while the GCG network shows that genes with similar properties form a large cluster and genes with different properties form a fragmented cluster.

## Discussion

To identify the characteristics of the GCG network we compared it with a similar GG network based on extracted gene entities from 331,411 articles in the field of bioinformatics. The constructed GG network consisted of 9,550 nodes and 55,610 edges, while the constructed GCG network consisted of 7,947 nodes, and 77,110 edges. Of the top 25 ranked genes in the GG network, all were also found in BioGRID. Within the GCG network, 96% of the top 25 ranked genes were found in BioGRID. This compares favorably with the accuracy measurement of other co-occurrence studies. Stephens et al. [7] evaluated their network against a Molecular Biology textbook [26], and ER transport pathway related genes for Golgi to achieve 67% and 50% accuracy respectively. Jensen et al. [6] used randomly selected pairs to evaluate their network against DIP and OMIM to find a 51% accuracy rate against DIP and 45% accuracy rate against OMIM. 

We analyzed both the GG network and the GCG network using the following measures: degree, weighted degree, closeness, betweenness, and PageRank. We observed that the top ranked genes are similar in both networks for each measure. The percent of genes identified in both networks by degree, weighted degree, closeness, betweenness, and PageRank is 33%, 64%, 0%, 36%, and 56% respectively. Combining all genes by these measures, results in 33% of genes appearing both networks. We identified disease clusters based on genes by consulting with the GAD DB and found the majority of genes relate to the cancer.

Overall, we observed no significant difference between the GG network and the GCG network, which indicates gene interaction through citation analysis, could be a novel approach to extracting gene-gene interaction from scientific literature. The basic assumption of citation analysis is that there is a subject relationship between two papers; since the GCG network utilizes citation relation to find gene interaction, it assumes that gene-gene pairs have topical, implicit relationships. Therefore, the GCG network can be used as a tool to analyze gene interaction in an implicit manner, which is particularly useful for a study that aims to extract novel gene relations. 

We examined whether top ranked gene pairs had known interactions by matching with BioGRID; revealing a matching rate of 28.03% in the GG network and 18.15% in the GCG network. This indicates that the GCG network may be less effective alone for detecting gene interactions. If we combine top ranked genes in both networks, the matching rate increases to 35.53%, indicating the GCG network can augment the existing the GG networks. 

There were 1,344 gene pairs identified in the GCG network, but not in the GG network, and which have known gene interactions in BioGRID. Among these pairs, there were five gene pairs with the number of co-occurrences over 100. These pairs are HYOU1-SIL1, EWSR1-TDRD3, DDX3X-TDRD3, CDK7-GTF2H5, and AXIN1-LRP5. We then examined these pairs against four well-known bio-entity databases: BioGraph (http://biograph.be), CTD (http://ctdbase.org), pharmGKB (http://www.pharmgkb.org), and GeneCards (http://www.genecards.org). PharmGKB, revealed no interaction information among these pairs. However, BioGraph, showed a high interaction between CDK7 and GTF2H5. According to the BioGraph knowledge base (http://biograph.be/project/project), the GTF2H5 gene is ranked first out of 18180 gene concepts (top 0.01%), in relation to the CDK7 gene, the LRP5 gene is ranked 18th in relation to AXIN1, the SIL1 gene is ranked 37th in relation to HYOU1, the TDRD3 gene is ranked 6492nd in relation to EWSR1 and 5235th in relation to DDX3X. 

In the CTD the interaction type between HYOU1 and SIL1 is marked as genetic and their throughput is low. This interaction is described by Zhao et al. [27] with the findings that the overexpression of HYOU1 with SIL1 reduces ER stress and rescues neuro-degeneration in Sil1(-/-) mice. Goulet et al. [28] reports the interaction between TDRD3 and EWSR1 as well as TDRD3 and DDX3X is physical and their throughput is low. Giglia-Mari et al. [29] reports the interaction between CDK7 and GTF2H5 is also physical with a low throughput. Four papers [30-33] reported the interaction between AXIN1 and LRP5 as physical with a low throughput. Table 6 summarizes these results, which shows that various diseases associated with gene-gene pairs identified only in the GCG network are worthy of investigation as to whether there exists direct, explicit interaction between genes. The implicit relationship among genes using the GCG network may thus provide a potential research direction in bioinformatics.

**Table 6:** Top 20 gene pairs detected only in GCG network.

**Gene-Gene Pair**	**Pair Occurrence**	**Gene object ranking in BioGraph (top %)**	**CTD interaction information**		
			**Interaction Type**	**Throughput**	**Article**
HYOU1-SIL1	350	37 (0.20%)	Genetic	low	**Zhao L, et al. (2010)**
EWSR1-TDRD3	176	6,492 (35.71%)	Physical	low	Goulet I, et al. (2008)
DDX3X-TDRD3	168	5,235 (28.80%)	Physical	low	Goulet I, et al. (2008)
CDK7-GTF2H5	152	1 (0.01%)	Physical	low	Giglia-Mari G, et al. (2004)
AXIN1-LRP5	124	18 (0.10%)	Physical	low	Kim MJ, et al. (2008).
			Physical	low	**Haÿ E, et al. (2009)**
			Physical	low	**Mao J, et al. (2001)**
			Physical	low	**Ding Y, et al. (2008)**

In addition, we conducted another experiment to investigate whether the GCG network revealed novel gene-gene interaction compared to the GG network. We selected gene-gene pairs that do not appear in the GG network but do appear in the GCG network only within a certain time-period. If those gene-gene pairs appear in the GG network after the given time-period, it indicates that researchers have studied direct gene-gene interaction between these two genes. Since the articles used to build networks were published between 2000 and 2011, we divided the data into two sets; 2000-2005 and 2006-2011. Then, we built the GG and the GCG networks with data set from 2000 to 2005; we found 37,658 gene pairs that appear only in the GCG network. Among these pairs, 1,149 pairs had confirmed gene-gene interaction based on BioGRID. A total of 164 pairs out of 1,149 were found in the GG network that was built using the entire data collection. This means that the 164 gene pairs that were not found in the GG network before 2005 were newly studied since then. In particular, the PARK2 and PINK1 gene pair ranks fifth by co-occurrence frequency in the GG network, implying the gene pair has highly been studied since 2005. Table 7 lists the gene-gene pairs with more than 100 co-occurrence frequencies appearing in the GCG network before 2005. 

**Table 7:** Gene-gene pairs with more than 100 co-occurrence frequencies in the GCG network by year 2005.

**Gene-Gene Pair**	**2005_GCG Pair Occurrence**	**ALL_GG Pair Occurrence frequency (Rank)**	**Gene object ranking in BioGraph (top %)**
PARK2 - PINK1	352	1726 ( 5 )	164 (0.23%)
ARF1 - RAB5A	167	18 ( 6092)	155 (0.22%)
DVL1 - LRRK2	126	24 ( 4425 )	386 (0.54%)
DLX5 - TP63	125	33 ( 3023)	242 (0.34%)
MDM2 - SENP2	122	12 ( 9111 )	5459 (7.69%)
MCPH1 - RAD51	117	60 ( 1320 )	15 (0.02%)
EP300 - SIRT1	114	52 ( 1668 )	306 (0.43%)

## Conclusion

In the present study, we explored implicit gene interaction through a GCG network. Unlike the GG network, which identifies direct gene relation based on gene co-occurrence, the GCG network identifies indirect relation based on citation. The results show that the GCG network shares many genes with the GG network and as a result is a competitive complement to the GG network, despite having slightly less accuracy that GG network in comparison with BioGRID. 

We have demonstrated that using gene relationships based on citation relation extends the assumption of gene interaction being limited to the same article and opens up a new opportunity to analyze gene interaction from a wider spectrum of datasets. In the present study, we examined only one link of citation relation, however in future work we intend to examine the chain of citation relation and apply co-citation analysis to the GCG network. 

The GCG network is proven useful for detecting gene interaction in an implicit manner, thus, confirming that the entitymetrics model proposed by Ding et al. [2] can be used to analyze gene relationships and other bio types such as disease or protein, and possibly applied to a heterogeneous network such as gene-disease or protein-organ.

## Supporting Information

Appendix S1 **Table A: Top 25 genes by degree centrality and associated disease categories in both network.** Table B: Top 25 genes by betweenness centrality and associated disease categories in both network. Table C: Top 25 genes by Pagerank and associated disease categories in both network. Table D: In Gene-Gene network, associated disease and representative gene in each cluster. Table E: In Gene-Citation-Gene network, associated disease and representative gene in each cluster.
(DOCX)
