# Correction: Discovering Implicit Entity Relation with the Gene-Citation-Gene Network

**DOI:** 10.1371/annotation/b6121731-7357-4657-9845-82eb0c937f89

**Published:** 2014-01-07

**Authors:** Min Song, Nam-Gi Han, Yong-Hwan Kim, Ying Ding, Tamy Chambers

The number of a grant in the Funding statement is incorrect. In addition, funding organizations are incorrectly omitted from the Funding statement. The Funding statement should read: 

"This research was supported by the Bio & Medical Technology Development Program of the National Research Foundation (NRF) funded by the Ministry of Science, ICT & Future Planning (Grant No. 2013M3A9C4078138)."

