# Supplementary material for: Discovering Implicit Entity Relation with the Gene-Citation-Gene Network
Source: PLoS One. 2013 Dec 17;8(12):e84639. doi: 10.1371/journal.pone.0084639 (PMC3866152; doi:10.1371/journal.pone.0084639)
Supplement: Appendix S1 — Table A: Top 25 genes by degree centrality and associated disease categories in both network. Table B: Top 25 genes by betweenness centrality and associated disease categories in both network. Table C: Top 25 genes by Pagerank and associated disease categories in both network. Table D: In Gene-Gene network, associated disease and representative gene in each cluster. Table E: In Gene-Citation-Gene network, associated disease and representative gene in each cluster. (DOCX) [file pone.0084639.s001.docx]

**Supporting Information tables**

Table A.Top 25 genes by degree centrality and associated disease categories in both network

| **Gene-Gene Network** | | | **Gene-Citation-Gene Network** | | |
| --- | --- | --- | --- | --- | --- |
| *Gene* | *Degree* | *Disease by GAD* | *Gene* | *Degree* | *Disease by GAD* |
| TP53 | 55 | CANCER ( 360 ) | TP53 | 66 | CANCER ( 360 ) |
| CLOCK | 18 | PSYCH ( 16 ) | RAD51 | 23 | CANCER ( 68 ) |
| MYC | 15 | CANCER ( 27 ) | SOX2 | 19 | VISION ( 5 ) |
| RAD51 | 14 | CANCER ( 68 ) | MYC | 17 | CANCER ( 27 ) |
| TERF2IP | 13 | CANCER ( 3 ) | CLOCK | 16 | PSYCH ( 16 ) |
| E2F1 | 12 | CANCER ( 3 ) | RAD9A | 14 | CANCER ( 1 ) |
| RAC1 | 10 | CANCER ( 4 ) | PLK1 | 13 | CANCER ( 6 ) |
| PLK1 | 10 | CANCER ( 6 ) | CTCF | 12 | METABOLIC ( 3 ) |
|  |  |  | DMC1 | 12 | REPRODUCTION ( 2 ) |
|  |  |  | MDM2 | 11 | CANCER ( 126 ) |
|  |  |  | PINK1 | 11 | NEUROLOGICAL ( 39 ) |
|  |  |  | PARK2 | 10 | NEUROLOGICAL ( 82 ) |
|  |  |  | SIRT1 | 10 | METABOLIC ( 6 ) |
|  |  |  | BUB1 | 10 | CANCER ( 5 ) |
|  |  |  | PAX6 | 10 | VISION ( 7 ) |

Table B.Top 25 genes by betweenness centrality and associated disease categories in both network

| **Gene-Gene Network** | | | **Gene-Citation-Gene Network** | | |
| --- | --- | --- | --- | --- | --- |
| *Gene* | *Betweenness* | *Disease by GAD* | *Gene* | *Betweenness* | *Disease by GAD* |
| TP53 | 61080.40714 | CANCER ( 360 ) | TP53 | 77397.18907 | CANCER ( 360 ) |
| MYC | 18594.83095 | CANCER ( 27 ) | MYC | 24435.49472 | CANCER ( 27 ) |
| PLK1 | 13893.23333 | CANCER ( 6 ) | C21orf33 | 17408.68564 | IMMUNE ( 2 ) |
| CLOCK | 10563.98333 | PSYCH ( 16 ) | RAD51 | 13447.71633 | CANCER ( 68 ) |
| RAD51 | 10191.5 | CANCER ( 68 ) | TFAP2A | 11167.92378 | * |
| SOX2 | 8532.066667 | VISION ( 5 ) | NOTCH1 | 10108.79444 | CANCER ( 11 ) |
| NR4A1 | 7744.016667 | NEUROLOGICAL ( 2 ) | SOX2 | 9703.547487 | VISION ( 5 ) |
| SMAD3 | 7666.280952 | CARDIOVASCULAR ( 9 ) | PLK1 | 9187.482806 | CANCER ( 6 ) |
| TERF2IP | 6407.183333 | CANCER ( 3 ) | FOLH1 | 9056 | * |
| PAX6 | 6400 | VISION ( 7 ) | SIRT1 | 8775.609802 | METABOLIC ( 6 ) |
| RHOD | 6351 | VISION ( 9 ) | YY1 | 8177.713745 | CHEMDEPENDENCY ( 1 ) |
| WWOX | 6265.5 | CARDIOVASCULAR ( 22 ) | CTCF | 7182.018507 | METABOLIC ( 3 ) |
| CTCF | 6005 | METABOLIC ( 3 ) | CLOCK | 6599 | PSYCH ( 16 ) |
| TP53BP1 | 6003.333333 | CANCER ( 11 ) | GRN | 6089 | NEUROLOGICAL ( 26 ) |
| WBP4 | 5607.5 | * | MLL | 6004.725599 | CANCER ( 13 ) |
| KLF4 | 4875 | CARDIOVASCULAR ( 4 ) | RAD9A | 5528.257878 | CANCER ( 1 ) |
| BUB1B | 4786.75 | CANCER ( 3 ) | SENP2 | 5276 | * |
| HDAC1 | 4771.638095 | CANCER ( 3 ) | ARX | 5274 | DEVELOPMENTAL ( 12 ) |
| RAC1 | 4567 | CANCER ( 4 ) | POU2F1 | 5232 | METABOLIC ( 3 ) |
| SETD2 | 4564 | PSYCH ( 1 ) | PAX6 | 4840.720715 | VISION ( 7 ) |
| SOCS3 | 4557 | METABOLIC ( 3 ) | SGK1 | 4835 | METABOLIC ( 6 ) |
| MITF | 4409.540476 | METABOLIC ( 4 ) | BCL2 | 4772.968519 | CANCER ( 33 ) |
| RAD9A | 4306 | CANCER ( 1 ) | PINK1 | 4727.546632 | NEUROLOGICAL ( 39 ) |
| E2F1 | 3903.45 | CANCER ( 2 ) | RHOD | 4171.771987 | VISION ( 9 ) |
| PTEN | 3789 | CANCER ( 49 ) | FGF8 | 3995.696807 | METABOLIC ( 1 ) |

Table C.Top 25 genes by Pagerank and associated disease categories in both network

| Gene-Gene Network | | | Gene-Citation-Gene Network | | |
| --- | --- | --- | --- | --- | --- |
| *Gene* | *PageRank* | *Disease by GAD* | *Gene* | *PageRank* | *Disease by GAD* |
| TP53 | 0.021952609 | CANCER ( 360 ) | TP53 | 0.029983791 | CANCER ( 360 ) |
| MDM2 | 0.006959965 | CANCER ( 126 ) | MDM2 | 0.00983107 | CANCER ( 126 ) |
| CLOCK | 0.00539187 | PSYCH ( 16 ) | CLOCK | 0.00876727 | PSYCH ( 16 ) |
| RAD51 | 0.004659456 | CANCER ( 68 ) | RAD51 | 0.007445446 | CANCER ( 68 ) |
| POU5F1 | 0.004434127 | IMMUNE ( 10 ) | SOX2 | 0.006660122 | VISION ( 5 ) |
| SOX2 | 0.004078862 | VISION ( 5 ) | PINK1 | 0.00520839 | NEUROLOGICAL ( 39 ) |
| RAC1 | 0.003865621 | CANCER ( 4 ) | MYC | 0.004869966 | CANCER ( 27 ) |
| TERF2IP | 0.003578382 | CANCER ( 3 ) | TP63 | 0.004541513 | CANCER ( 11 ) |
| MYC | 0.003506265 | CANCER ( 27 ) | MLL | 0.004465544 | CANCER ( 13 ) |
| RAB5A | 0.00319853 | * | NOX4 | 0.004185311 |  |
| E2F1 | 0.002994979 | CANCER ( 2 ) | PLK1 | 0.004016882 | CANCER ( 6 ) |
| PINK1 | 0.002966762 | NEUROLOGICAL ( 39 ) | POU5F1 | 0.00400632 | IMMUNE ( 10 ) |
| SOX9 | 0.002836915 | METABOLIC ( 4 ) | PARK2 | 0.003981217 | NEUROLOGICAL ( 82 ) |
| NANOG | 0.002773498 | CARDIOVASCULAR ( 1 ) | CTCF | 0.00396477 | METABOLIC ( 3 ) |
| MYD88 | 0.002638534 | IMMUNE ( 4 ) | RAD9A | 0.003789082 | CANCER ( 1 ) |
| MXI1 | 0.002625402 | IMMUNE ( 2 ) | DMC1 | 0.003728564 | REPRODUCTION ( 2 ) |
| UPF1 | 0.00260818 | * | PAX6 | 0.003529091 | VISION ( 7 ) |
| CTCF | 0.002555002 | METABOLIC ( 3 ) | PAX3 | 0.003440291 | AGING ( 2 ) |
| SETD2 | 0.002536053 | PSYCH ( 1 ) | BCL2 | 0.003416004 | CANCER ( 33 ) |
| PARK2 | 0.002422325 | NEUROLOGICAL ( 82 ) | TXN | 0.00337162 | NEUROLOGICAL ( 51 ) |
| PLK1 | 0.002401844 | CANCER ( 6 ) | TP73 | 0.003341228 | CANCER ( 28 ) |
| DMC1 | 0.002399675 | REPRODUCTION ( 2 ) | TDRD3 | 0.003318872 |  |
| CDKN2A | 0.002384565 | CANCER ( 131 ) | RPGR | 0.003284319 | VISION ( 6 ) |
| CDC20 | 0.002368066 | CANCER ( 2 ) | MDM4 | 0.003220019 | CANCER ( 6 ) |
| BCL2 | 0.002318147 | CANCER ( 33 ) | RAB5A | 0.003178761 | * |

Table D.In Gene-Gene network, associated disease and representative gene in each cluster

| **Cluster** | **Representative Genes** | **Disease** |
| --- | --- | --- |
|  |  | **( no. of genes studied for the disease in the cluster) / (total no. of genes in the cluster))** |
| 1 | RAG1, RAG2 | Immunologic deficiency and Lymphoma diseases |
|  |  | Lymphoma, Non-Hodgkin 2 / 2 |
|  |  | Severe Combined Immunodeficiency 2 / 2 |
|  |  | Multiple Myeloma 2 / 2 |
|  |  | Immunologic Deficiency Syndromes 2 / 2 |
|  |  | SCID 2 / 2 |
|  |  | Lymphopenia 2 / 2 |
| 2 | RHOD, RAC1 | Various diseases including tobacco use disorder, amyotrophic lateral sclerosis, and schizophrenia |
|  |  | Tobacco Use Disorder 4 / 16 |
|  |  | Amyotrophic Lateral Sclerosis 3 / 16 |
|  |  | Schizophrenia 3 / 16 |
|  |  | Alzheimer's disease 3 / 16 |
|  |  | chronic obstructive pulmonary disease 3 / 16 |
|  |  | bladder cancer 3 / 16 |
|  |  | Cholesterol, LDL 3 / 16 |
|  |  | lung cancer 3 / 16 |
|  |  | Leukemia 3 / 16 |
| 3 | CTCF, DNMT3B, DNMT3A, DNMT1, H19 | Breast and epithelial ovarian cancers |
|  |  | breast cancer 5 / 12 |
|  |  | epithelial ovarian cancer 4 / 12 |
|  |  | subtelomeric hypomethylation 4 / 12 |
|  |  | colorectal cancer 3 / 12 |
|  |  | arsnic exposure 2 / 12 |
|  |  | Stomach Neoplasms 2 / 12 |
|  |  | healthy oldest-old 2 / 12 |
|  |  | bladder cancer 2 / 12 |
|  |  | benzo[a]pyrene diol epoxide 2 / 12 |
|  |  | Lipoproteins, HDL 2 / 12 |
|  |  | Spinal Dysraphism 2 / 12 |
|  |  | lung cancer 2 / 12 |
|  |  | hunger and satiety 2 / 12 |
| 4 | EIF2C2, FMR1, FXR1 | Kidney, oral, and esophageal diseases |
|  |  | Kidney Neoplasms 2 / 7 |
|  |  | esophageal cancer 2 / 7 |
|  |  | Carcinoma, Renal Cell 2 / 7 |
|  |  | oral premalignant lesions 2 / 7 |
|  |  | lung cancer 2 / 7 |
| 5 | TP53, HRAS, MYC, BCL2, EP300, CDKN2A | Various diseases including breast and lung cancers and neoplasms |
|  |  | Tobacco Use Disorder 19 / 70 |
|  |  | lung cancer 19 / 70 |
|  |  | breast cancer 18 / 70 |
|  |  | colorectal cancer 16 / 70 |
|  |  | Pancreatic Neoplasms 16 / 70 |
| 6 | RAD51, TERF2IP, MRE11A | Various cancer including breast cancer, bladder cancer |
|  |  | breast cancer 13 / 28 |
|  |  | bladder cancer 9 / 28 |
|  |  | lung cancer 9 / 28 |
|  |  | ovarian cancer 7 / 28 |
|  |  | epithelial ovarian cancer 7 / 28 |
| 7 | MXI1, CDC20, MXD1, BUB1, BUB3, BUB1B | Cancer and neurological disease |
|  |  | breast cancer 5 / 9 |
|  |  | ovarian cancer 2 / 9 |
|  |  | Alcoholism 2 / 9 |
|  |  | Body Height 2 / 9 |
| 8 | CLOCK, ARNTL, PER2 | Prostate cancer and neurological diseases including depression, schizophrenia |
|  |  | prostate cancer 7 / 11 |
|  |  | depression 7 / 11 |
|  |  | bipolar disorder 7 / 11 |
|  |  | Sleep Disorders 7 / 11 |
|  |  | schizophrenia 6 / 11 |
| 9 | HHEX, FTO, IGF2BP2, CDKAL1, SLC30A8 | Calcinosis, HIV, obesity, and diabetes diseases |
|  |  | Calcinosis 5 / 5 |
|  |  | HIV Infections 5 / 5 |
|  |  | atherosclerosis 5 / 5 |
|  |  | Diabetes Mellitus 5 / 5 |
|  |  | [X]Human immunodeficiency virus disease 5 / 5 |
|  |  | obesity 5 / 5 |
|  |  | BMI 5 / 5 |
|  |  | Diabetes mellitus type II 5 / 5 |
|  |  | reduced prostate cancer risk 5 / 5 |
|  |  | Coronary Artery Disease 5 / 5 |
|  |  | diabetes, type 1 5 / 5 |
|  |  | Insulin Resistance 5 / 5 |
| 10 | PAX6, FGF8, OTX2, AES | Eye |
|  |  | Coloboma 3 / 10 |
|  |  | Retinal Diseases 3 / 10 |
|  |  | microphthalmia 3 / 10 |
|  |  | Retinitis Pigmentosa 2 / 10 |
|  |  | Body Mass Index 2 / 10 |
|  |  | Eye Abnormalities 2 / 10 |
| 11 | HDAC2, KAT2B | Various diseases including tobacco use disorder and schizophrenia |
|  |  | Tobacco Use Disorder 3 / 17 |
|  |  | schizophrenia 2 / 17 |
|  |  | Amyotrophic Lateral Sclerosis 2 / 17 |
| 12 | HSPD1, HSPE1 | Various diseases including spastic paralysis and Rheumatoid Arthritis |
|  |  | spastic paralysis 1 / 2 |
|  |  | Rheumatoid Arthritis 1 / 2 |
|  |  | ovarian cancer 1 / 2 |
|  |  | Coronary Disease 1 / 2 |
|  |  | Diabetes Mellitus 1 / 2 |
|  |  | Alzheimer's disease 1 / 2 |
| 13 | SMN1, SMN2 | Muscular atrophy disease |
|  |  | ALS/amyotrophic lateral sclerosis 2 / 2 |
|  |  | Spinal Muscular Atrophies of Childhood 2 / 2 |
|  |  | Spinal Muscular Atrophy 2 / 2 |
|  |  | Muscular Atrophy, Spinal 2 / 2 |
|  |  | Motor Neuron Disease 2 / 2 |
| 14 | HOXC8, HOXD8, HOXB8 | Clubfoot and bone mineral density diseases |
|  |  | Clubfoot 1 / 3 |
|  |  | Bone Mineral Density 1 / 3 |
| 15 | MAP3K7, MYD88, DDX58, IFIH1, TAB2, NOD2 | Autoimmune disease |
|  |  | Lupus Erythematosus, Systemic 3 / 6 |
|  |  | Encephalomyelitis, Autoimmune, Experimental 3 / 6 |
|  |  | Multiple Sclerosis 3 / 6 |

Table E.In Gene-Citation-Gene network, associated disease and representative gene in each cluster

| **Cluster** | **Representative Genes** | **Disease** |
| --- | --- | --- |
|  |  | **( no. of genes studied for the disease in the cluster) / (total no. of genes in the cluster))** |
| 1 | NOD2, CA9, ERAP1, ERAP2 | Pre-eclampsia and spondylitis diseases |
|  |  | Pre-Eclampsia 3 / 4 |
|  |  | Spondylitis, Ankylosing 3 / 4 |
|  |  | Psoriasis 2 / 4 |
|  |  | Crohn Disease 2 / 4 |
|  |  | Rheumatoid spondylitis 2 / 4 |
|  |  | Pregnancy Complications 2 / 4 |
|  |  | ankylosing spondylitis 2 / 4 |
|  |  | Hypertension 2 / 4 |
|  |  | Inflammation 2 / 4 |
|  |  | diabetes, type 1 2 / 4 |
| 2 | RAB5A, RAB7A, RAB8A | Various diseases including tuberculosis, abortion, and spontaneous |
|  |  | Tuberculosis 2 / 11 |
|  |  | Tobacco Use Disorder 2 / 11 |
|  |  | Abortion, Spontaneous 2 / 11 |
|  |  | Alcoholism 2 / 11 |
|  |  | inflammatory bowel disease 2 / 11 |
| 3 | TP53, HRAS, MYC, CDKN2A, BCL2, POU5F1, MARK2, PTEN, SOX2, MDM2 | Cancer and neoplasms diseases |
|  |  | Tobacco Use Disorder 24 / 109 |
|  |  | ovarian cancer 17 / 109 |
|  |  | prostate cancer 17 / 109 |
|  |  | Bone Mineral Density 15 / 109 |
|  |  | breast cancer 15 / 109 |
|  |  | lung cancer 15 / 109 |
| 4 | CTCF, SIRT1, NIPBL, DNMT1, DNMT3B | Obesity and various cancers |
|  |  | healthy oldest-old 5 / 19 |
|  |  | Obesity 4 / 19 |
|  |  | subtelomeric hypomethylation 4 / 19 |
|  |  | breast cancer 4 / 19 |
|  |  | Body Weight 4 / 19 |
|  |  | Diabetes Mellitus, Type 2 4 / 19 |
|  |  | epithelial ovarian cancer 4 / 19 |
| 5 | PRNP, PRND, SPRN | Brain related diseases including Creutzfeld-Jakob and Alzheimer's disease |
|  |  | Creutzfeld-Jakob disease 2 / 3 |
|  |  | Creutzfeldt-Jakob Syndrome 2 / 3 |
|  |  | Alzheimer's disease 2 / 3 |
|  |  | encephalopathies, transmissible spongiform 2 / 3 |
|  |  | Tobacco Use Disorder 2 / 3 |
| 6 | SGK1, HFE, WBP4 | Blood related diseases and heart failure |
|  |  | Heart Failure 3 / 6 |
|  |  | Type 2 diabetes 2 / 6 |
|  |  | Respiratory Function Tests 2 / 6 |
|  |  | Pregnancy Complications 2 / 6 |
|  |  | Precursor Cell Lymphoblastic Leukemia-Lymphoma 2 / 6 |
|  |  | porphyria cutanea tarda 2 / 6 |
|  |  | Myeloproliferative Disorders 2 / 6 |
|  |  | mean corpuscular volume 2 / 6 |
|  |  | lung cancer 2 / 6 |
|  |  | Leukemia 2 / 6 |
|  |  | Iron Overload 2 / 6 |
|  |  | iron metabolism 2 / 6 |
|  |  | hypertension 2 / 6 |
|  |  | hepatocellular carcinoma 2 / 6 |
|  |  | hepatitis C 2 / 6 |
|  |  | hemochromatosis 2 / 6 |
|  |  | Erythrocyte Indices 2 / 6 |
|  |  | Diabetes Mellitus, Type 2 2 / 6 |
|  |  | colorectal cancer 2 / 6 |
|  |  | cirrhosis 2 / 6 |
|  |  | chronic obstructive pulmonary disease 2 / 6 |
|  |  | Cholesterol, LDL 2 / 6 |
|  |  | breast cancer 2 / 6 |
|  |  | bladder cancer 2 / 6 |
|  |  | Birth Weight 2 / 6 |
|  |  | Abortion, Spontaneous 2 / 6 |
| 7 | RAD51, RHOD, PLK1, RAD50, RAD9A, MRE11A, CDC20, CDC5L | Various cancers |
|  |  | breast cancer 19 / 44 |
|  |  | ovarian cancer 10 / 44 |
|  |  | bladder cancer 9 / 44 |
|  |  | lung cancer 9 / 44 |
|  |  | Pancreatic Neoplasms 8 / 44 |
|  |  | Adenocarcinoma 8 / 44 |
| 8 | NDE1, DISC1, PAFAH1B1, NDEL1 | Schizophrenia |
|  |  | Schizophrenia 4 / 4 |
|  |  | Alzheimer's disease 2 / 4 |
